# Per os infectivity of white spot syndrome virus (WSSV) in white-legged shrimp (*Litopenaeus vannamei*) and role of peritrophic membrane

**DOI:** 10.1186/s13567-016-0321-5

**Published:** 2016-02-29

**Authors:** Khuong Van Thuong, Vo Van Tuan, Wenfeng Li, Patrick Sorgeloos, Peter Bossier, Hans Nauwynck

**Affiliations:** Laboratory of Virology, Faculty of Veterinary Medicine, Ghent University, Salisburylaan 133, 9820 Merelbeke, Belgium; Laboratory of Aquaculture, Artemia Reference Center, Faculty of Bioscience Engineering, Ghent University, Rozier 44, 9000 Ghent, Belgium; Research institute for Aquaculture number 1, Dinhbang, Tuson, Bacninh Vietnam

## Abstract

As earlier observations on peroral infectivity of WSSV in white-legged shrimp are conflicting, here, a standardized peroral intubation technique was used to examine (i) the role of the physical composition of the viral inoculum and (ii) the barrier function of the PM. In a first experiment, the infectivity of a WSSV stock was compared by determining the SID_50_ by intramuscular injection, peroral inoculation or via feeding. The following titers were obtained: 10^8.77^ SID_50_/g by intramuscular injection, 10^1.23^ SID_50_/g by peroral inoculation and 10^0.73^ SID_50_/g by feeding. These results demonstrated that 10^7.54^–10^8.03^ infectious virus is needed to infect shrimp by peroral inoculation and via feeding. Next, it was examined if damage of the PM may increase the susceptibility for WSSV by peroral route. The infectivity of a virus stock was tested upon peroral inoculation of shrimp with and without removal of the PM and compared with the infectivity upon intramuscular inoculation. The virus titers obtained upon intramuscular injection and peroral inoculation of shrimp with and without PM were 10^8.63^, 10^1.13^ and 10^1.53^ SID_50_/mL, respectively. This experiment confirmed the need of 10^7.1^–10^7.5^ infectious virus to infect shrimp via peroral route and showed that the removal of the PM slightly but not significantly (*p* > 0.05) facilitated the infection of shrimp. This study indicated that WSSV contaminated feed is poorly infectious via peroral route, whereas it is highly infectious when injected into shrimp. The PM plays a minor role as internal barrier of shrimp against WSSV infection.

**Electronic supplementary material:**

The online version of this article (doi:10.1186/s13567-016-0321-5) contains supplementary material, which is available to authorized users.

## Introduction

Since its first description in 1992 [1], WSSV is responsible for a large number of failures of shrimp culture worldwide [2]. WSSV is a rod-shaped, enveloped virus that infects a broad range of crustaceans [3, 4]; to date, more than 90 crustacean species have been found as hosts or carriers of WSSV [5]. WSSV may be transmitted via vertical and horizontal routes [6–11]. Some environmental parameters such as temperature, salinity drop and pH are known as stressors influencing transmission and may influence the occurrence of WSSV outbreaks [12–16]. It is difficult to infect shrimp with WSSV via immersion or cohabitation with infected hosts [7, 8, 10, 17] and per os WSSV inoculation by intubation or via feed results in contradictory findings. Some researchers found it a powerful tool to induce WSSV infection in shrimp [16, 18, 19] whereas others had difficulties to reproduce these results [17, 20–22]. Differences in virulence of the WSSV strain, virus dose, way of administration, and experimental conditions of the animals may be responsible for these controversial observations.

The peritrophic membrane (PM) is a non-cellular structure, composed of chitin fibrils and proteins, which are synthesized and secreted by epithelial midgut cells. It is lining the epithelial midgut and acts as a barrier preventing animals from physical damages and pathogen invasion [23]. In insects, it is well known that the inhibition of PM formation may increase the susceptibility of the host to virus infection [24–28]. In order to establish an infection in the digestive tract of the host, pathogens may use their own chitinase to facilitate the penetration of PM [29, 30]. In shrimp, it was already demonstrated that bacteria such as *Vibrio parahaemolyticus* may destroy the PM barrier to initiate colonization and replication in the midgut and invasion in the shrimp body [31, 32]. In addition, prior to molting, shrimp increase the expression of endogenous chitinases, which may help in the degradation process of the PM and facilitate the pathogen invasion [33–36]. At present, it is not clear if the PM forms a barrier to WSSV and if a removal of the PM may help WSSV to infect midgut epithelial cells and invade into the shrimp.

In the present study, it was examined if the physical composition of the viral inoculum and presence/absence of the PM changes the capacity of WSSV to infect its host.

## Materials and methods

### Experimental animals and growing conditions

The shrimp used in this study were *Penaeus* (*Litopenaeus*) *vannamei* from Piti Syaqua Farm, Syaqua Siam Co. Ltd., Thailand. The batch of 10 000 PL_8-12_ was certified to be specific pathogen free (SPF) for the viruses WSSV, TSV, YHV and IHHNV by PCR and histopathology. The PL were transported to the Laboratory of Aquaculture and Artemia Reference Center (ARC), Ghent University, Belgium. At the ARC, shrimp were grown in a bio-filter recirculation system, fed with pelleted feed at a rate of 5% of mean body weight per day. Temperature was maintained at 27 ± 1 °C, salinity at 35 ± 1 g/L. Total ammonia and nitrite were controlled to be lower than 0.5 and 0.15 mg/L, respectively. For the inoculation experiments, shrimp were transported to the Laboratory of Virology, Faculty of Veterinary Medicine, Ghent University.

### Determination of molt stage

Based on the descriptions of Robertson et al. [37], Chan et al. [38] and Corteel et al. [39], the molt cycle of shrimp was determined and the shrimp were separated into 5 major stages. Briefly, shrimp were restrained and their exopodites of uropods were examined and analyzed on the appearance of setae, epidermis and cuticle under an inverted microscope at a magnification of 100×. In the early post-molt stage (A), the epidermis is present in the setae and retracts in later post-molt (B). In the inter-molt stage (C), the epidermis retracts under the setae and forms a straight line at the bottom of setae. In the early pre-molt (D1), the epidermis retracts from the old cuticle and starts forming a new cuticle. In the final stage, (before-molt, D2) new setae are formed under the old cuticle.

### WSSV preparation

#### Preparation of WSSV stock

A WSSV Thai-1 used in this study was collected from infected *P. monodon* in Thailand in 1996 and amplified in crayfish *Pacifastacus leniusculus* [40]. A homogenate of WSSV infected crayfish gills, kindly donated by P. Jiravanichpaisal and K. Soderhall (Uppsala University, Sweden), was inoculated in SPF *P. vannamei* juveniles to produce a starting WSSV stock. The median infectious titer of the stock was 10^6.6^ SID_50_/mL as determined by in vivo intramuscular titration [41].

#### Preparation of WSSV stocks (WSSV stock 1a and 1b and WSSV stock 2)

From the starting WSSV stock, a dilution of 10^−2^ was made in phosphate-buffered saline (PBS, pH 7.4) and injected intramuscularly into SPF *P. vannamei* juveniles to amplify the virus. Moribund shrimp were collected and confirmed to be WSSV positive by indirect immunoflourescence (IIF). Three inoculation stocks were prepared.

A. Stocks 1a and 1b: One hundred grams of moribund WSSV-infected shrimp were weighed and thawed. The shell, hepatopancreas and gut were removed and the remaining body was longitudinally cut into two parts. The first part was homogenized at 5000 rpm for 5 min using an IKA T 25 digital ultra-turrax. Then, the homogenate was further minced by serial syringe needles (1.2, 0.9 and 0.55 × 20 mm). Briefly, the homogenate was sucked up and blown out several times through the needle of 1.2 × 20 mm attached to a 20 mL syringe; this was repeated with needles of 0.9 × 20 mm and 0.55 × 20 mm, aliquoted and stored at −70 °C for intramuscular injection and peroral inoculation experiments (stock 1a). The second part of WSSV infected tissue was cut into small pieces of 0.5-1 mm^2^ and stored at −70 °C for feeding (stock 1b).
B. Stock 2: For the preparation of WSSV stock 2, 50 g of thawed shrimp without shell, hepatopancreas and gut were chopped, suspended in PBS at a ratio of 1:3, homogenized at 5000 rpm for 1–1.5 min using IKA T 25 digital ultra-turrax and centrifuged at 3500 rpm for 10 min (4 °C). Then, supernatant was collected and stored at −70 °C (WSSV stock 2).

### Experimental design

#### Effect of physical composition of the viral inoculum on the oral infectivity of WSSV

The aim of this experiment was to compare the infectivity of a WSSV stock by determining the SID_50_ by intramuscular and peroral inoculation of a viral suspension and via feeding of infected tissue from the same shrimp. In the experiment, early pre-molt (D1) *P. vannamei* juveniles (MBW = 4.86 ± 0.37 g, *n* = 210) were collected and acclimated individually for 24 h in 10-liter tanks. Then, one group of twenty shrimp was injected with 50 mg of a 10-fold serial dilution (10^−6^, 10^−7^, 10^−8^, 10^−9^, five animals per dilution) of WSSV stock 1a. Another group of twenty shrimp was inoculated perorally with 50 mg of a 10-fold serial dilution (10^0^, 10^−1^, 10^−2^, 10^−3^, five animals per dilution) of the same WSSV stock 1 a using a 0.74 × 19 mm—24G Surflo-W catheter (a 10-fold serial dilution was prepared by mixing 1 portion of WSSV stock with 9 portions of pathogen-free shrimp minced tissue). Thirty shrimp of the third group were naturally fed per os with 0.5, 5, 50, 100, 250 and 500 mg of WSSV chopped tissue, with 5 shrimp per dose. Shrimp were fed one meal for the dose of 0.5, 5, 50 and 100 mg, 3 and 5 meals for the feeding of 250 and 500 mg of WSSV chopped tissue, respectively. The time interval between the two meals was 1 h. After inoculation, shrimp were housed individually and kept for 5 days. Cephalothoraxes and midguts of dead and moribund shrimp were collected every 12 h and terminated at 120 hpi. Samples of cephalothoraxes and midguts of dead, moribund and euthanized shrimp at 120 hpi were processed for detection of WSSV infection by IIF. The experiment was performed three times.

#### Role of PM as intestinal barrier

##### Removal of the PM

In this experiment, it was aimed to remove the PM by a peroral flushing of the midgut. A total of 24 *P. vannamei* juveniles (MBW = 4.62 ± 0.68 g) were screened for their molt stages (B, C, D_1_, D_2_). Six shrimp were selected in each of the four major molt stages and divided into two groups. Shrimp of both groups were fed with pathogen-free shrimp chopped tissue. The animals were starved then for 4 h. Shrimp in the first group were given a peroral flush using 1 mL of PBS. This was done with a 1 mL syringe attached to a 24G Surflo-W catheter. The catheter was gently inserted inside the shrimp mouth chamber. By a gentle press on the plunger of the syringe, the PBS was forced through the shrimp digestive tract. Shrimp in the second group were not flushed.

##### Cryosection and staining of peritrophic membrane

**Figure 1:**
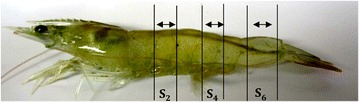
**Three sampling sites for analysis of the peritrophic membrane.** Segment S_2_ and S_4_ contain midgut, segment S_6_ contains hindgut.

After flushing, the fecal material that was expelled out of the anus was collected, fixed in 4% paraformaldehyde and incubated with 25 µg/mL fluorescein-linked succinylated WGA (wheat germ agglutinin lectin from *Triticum vulgarus*; Vector FL 1021S) for PM analysis. After flushing, shrimp of both groups were euthanized on ice and dissected (5 mm in length) at 3 sites (S_2_, S_4_, S_6_, see Figure 1). Dissected tissues were fixed in 4% paraformaldehyde at room temperature (22 °C) for 15 min, washed with PBS for 15 min, embedded in 2% methylcellulose and frozen in liquid nitrogen liquid for 8 min. Cryosections (5 µm) were made and mounted on slides, washed with PBS for 5 min, incubated with fluorescein-linked succinylated WGA (25 µg/mL) for 30 min and Hoechst (10 µg/mL) for 15 min. Then, the slides were washed twice with PBS and once in deionised water (3 min each), dried and mounted with glycerin DABCO. The slides were analyzed by fluorescence microscopy (Leica DM RBE) and microphotographs were made at 100× magnification.

##### Effect of PM removal on WSSV infection upon peroral inoculation with WSSV

The aim of this experiment was to evaluate the barrier function of PM to WSSV infection via oral route. In this experiment, early pre-molt (D1) *P. vannamei* juveniles (MBW = 4.55 ± 1 g, *n* = 110) were screened, housed individually in 10 liter-aquaria and acclimated for 24 h. Prior to inoculation, shrimp were fed before a starvation period of 4 h. Then, fifteen shrimp were injected intramuscularly with 50 μL of a 10-fold serial dilution (10^−6^–10^−8^, five animals per dilution) of WSSV stock 2. In twenty shrimp, the PM was removed by a peroral flush as described in sub-section “Removal of the PM” and twenty shrimp were kept intact. Afterwards, the animals were inoculated perorally with 50 μL of a 10-fold serial dilution (10^0^, 10^−1^, 10^−2^, 10^−3^, five animals per dilution) of the same WSSV stock 2. After inoculation, shrimp were housed individually and fed with commercial shrimp diet at a rate of 5% of mean body weight per day. Moribund and dead shrimp were recorded and removed from the aquaria every 12 h until the end of the experiment at 120 hpi. Cephalothoraxes and midguts of dead, moribund and surviving shrimp were processed for detection of WSSV infection by IIF. The whole experiment was repeated twice. Five shrimp were used per dilution in the first repeat. In the second repeat, fifteen shrimp were used per dilution.

### Detection of WSSV infection by indirect immunofluorescence (IIF)

WSSV infected shrimp were evaluated by indirect immunofluorescence (IIF) based on the description of Escobedo-Bonilla et al. [41]. Briefly, cephalothoraxes of dead, moribund and euthanized shrimp were collected, embedded in 2% methylcellulose and frozen at **−**20 °C. Cryosections (6 µm) were made and fixed for 10 min in 100% methanol at **−**20 °C. The sections were washed three times in PBS (5 min each) and were incubated with 2 µg/mL of monoclonal antibody 8B7 (Diagxotics Inc.USA) directed against viral protein VP28 for 1 h at 37 °C. Then, samples were washed three times in PBS (5 min each), incubated with fluorescein isothiocyanate (FITC)-labelled goat anti-mouse IgG (F-2761, Molecular Probes, The Netherlands) for 1 h at 37 °C. The cell nuclei were stained by Hoechst for 15 min. Finally, the samples were washed twice with PBS, rinsed once in deionised water, dried and mounted with a solution of glycerine and 1,4-diaza-bicyclo-octane (DABCO) (ACROS organics, USA). Sections were analyzed by fluorescence microscopy (Leica DM RBE).

### Statistical analysis

Virus infection titers (SID50) (Sub-sections “Effect of physical composition of the viral inoculum on the oral infectivity of WSSV” and “Effect of PM removal on WSSV infection upon peroral inoculation with WSSV”) were calculated based on the method of Reed and Muench [42]. Briefly, the numbers of infected and uninfected shrimp in each dilution were recorded. Accumulated values for the total number of animals that were infected or uninfected were obtained by adding values in the direction of the lowest to the highest values. The ratio and percentage of accumulated infected animals on the sum of the accumulated infected and uninfected animals were calculated for each dilution.

Two adjacent values, with one above (a) and one below (b) 50% were selected to calculate the proportional distance to the 50% endpoint by the following formula: (a-50%)/(a–b). The proportional distance was added to the log10 of the dilution, that contained the percentage above 50% (a). The value of shrimp infectious dose 50% endpoint (SID_50_) per mL was calculated taking into account to volume of the inoculum.

Shrimp of 3 replicates (Sub-section “Effect of PM removal on WSSV infection upon peroral inoculation with WSSV”) were pooled into 2 groups (100 shrimp with and 100 shrimp without removal of the peritrophic membrane). The difference in infection rates between 2 groups was tested by Pearson’s Chi square test. All calculations were performed using R version 3.1.2.

## Results

### Effect of physical composition of the viral inoculum on the oral infectivity of WSSV

**Figure 2:**
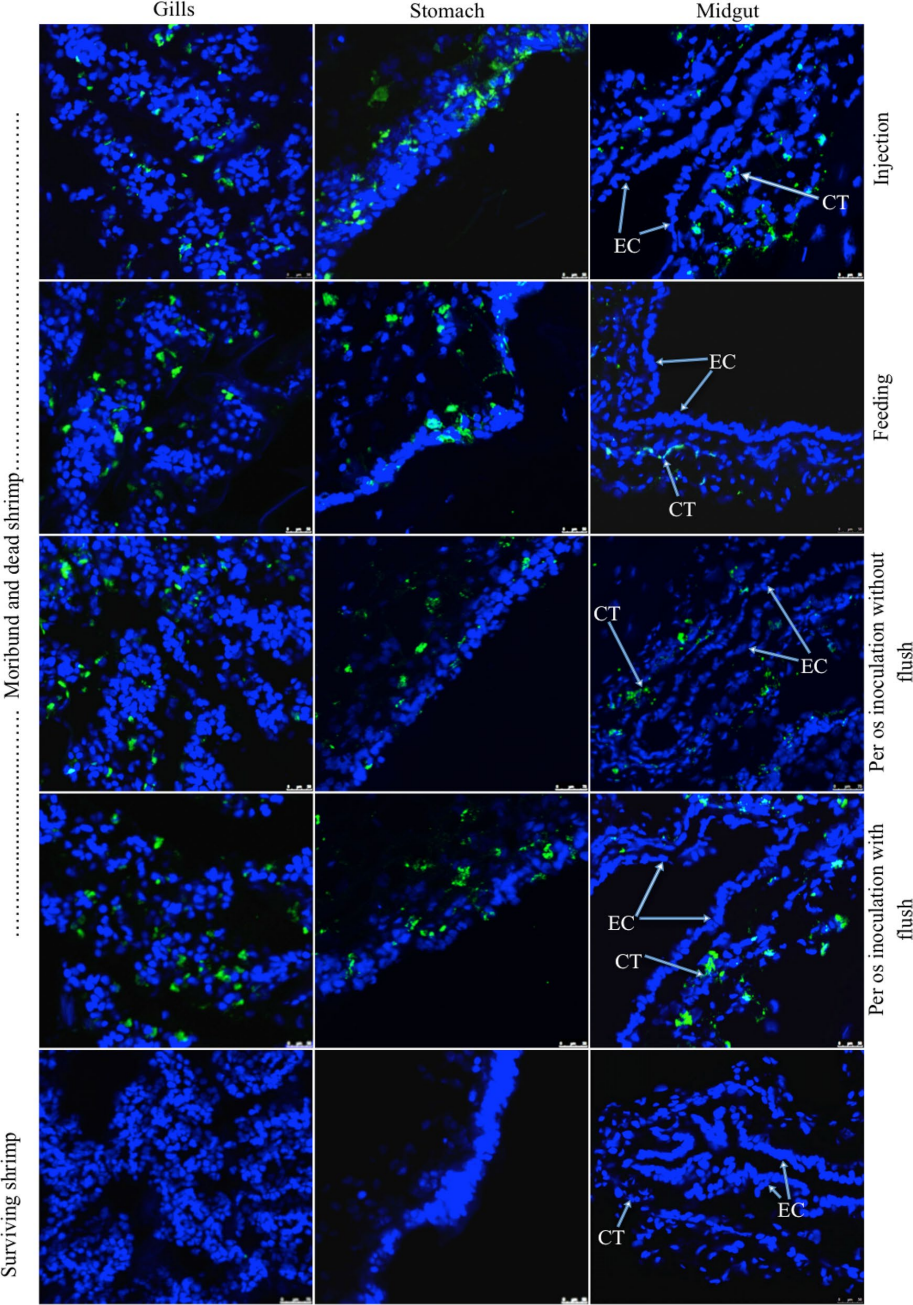
**Representavive photomicrographs of viral antigen positive cells (green) in different tissues of moribund and dead shrimp.** WSSV was detected by IIF using a VP28-specific mouse monoclonal antibody and an FITC-conjugated goat-anti mouse IgG. The cells of connective tissue (CT) of the midgut were WSSV positive, while the epithelial cells (EP) were WSSV negative.

Shrimp inoculated intramuscularly with WSSV concentrations 10^−6^–10^−8^ of stock 1a (WSSV suspension finely minced with needles) had a mortality of 5 out of 5, 4 out of 5 and 1 out of 5, respectively. All shrimp inoculated with dilution 10^−9^ survived until 120 hpi. The same results were obtained in the second experiment. In the third repeat, 5 out of 5 shrimp in the dilution 10^−6^ and 4 in the dilution 10^−7^ died. Other shrimp survived until the end of the experiment. Upon oral inoculation with the dilutions 10^0^, 10^−1^, 10^−2^ and 10^−3^ of the same WSSV stock 1a, only 2, 3, and 2 out of 5 shrimp inoculated with the dilution 10^0^ died in the three different repeats. When peroral feeding was performed with 0.5, 5, 50, 100, 250 and 500 mg of WSSV chopped tissue 1b, 0, 1, 0, 1, 2 and 3 animals out of 5 shrimp died, respectively, in the first experiment, 0, 0, 1, 2, 3 and 4 in the second experiment and 0, 0, 1, 2, 2 and 4 in the third repeat. IIF analysis of cephalothorax of dead, moribund and surviving shrimp revealed that all dead and moribund animals were WSSV positive, while all surviving shrimp were WSSV negative. Analysis of midgut of dead and moribund shrimp showed that connected tissue (CT) of the midgut of dead and moribund shrimp were WSSV positive, whereas epithelial cells (EC) were WSSV negative (Figure 2). The mean virus titers determined upon intramuscular injection, peroral inoculation and by feeding WSSV infected tissue were 10^8.76 ± 0.06^, 10^1.23 ± 0.23^ and 10^0.73 ± 0.12^ SID_50_ g^−1^, respectively (Table 1). Compared with the intramuscular route, 10^7.53^ times more virus was needed to infect a shrimp via oral inoculation, while 10^8.03^ times more virus was necessary to infect a shrimp via peroral feeding.

**Table 1:** **Determination of virus titers of a WSSV stock in** ***P. vannamei*** **by different inoculations.**

Experiment	Inoculation route	Dilution on homogenate of tissue	No. of shrimp	Mortality at different time points (hpi)									Infection by IIF	Virus titer
				24	36	48	60	72	84	96	120	Total		
1	Intramuscular	10^−7^	5		1	1	1		1			4/5	4/5	10^8.8^SID_50_/g
		10^−8^	5				1					1/5	1/5	
		10^−9^	5									0/5	0/5	
	Peroral	10^0^	5			1	1					2/5	2/5	10^1.1^SID_50_/g
		10^−1^	5									0/5	0/5	
		10^−2^	5									0/5	0/5	
		10^−3^	5									0/5	0/5	
	Feed	0.5	5									0/5	0/5	10^0.6^SID_50_/g
		5	5		1							1/5	1/5	
		50	5									0/5	0/5	
		100	5		1							1/5	1/5	
		250	5		1	1						2/5	2/5	
		500	5					3				3/5	3/5	
2	Intramuscular	10^−6^	5		1	3		1				5/5	5/5	10^8.8^SID_50_/g
		10^−7^	5			1	2	1				4/5	4/5	
		10^−8^	5				1					1/5	1/5	
		10^−9^	5									0/5	0/5	
	Peroral	10^0^	5				2	1				3/5	3/5	10^1.5^SID_50_/g
		10^−1^	5									0/5	0/5	
		10^−2^	5									0/5	0/5	
		10^−3^	5									0/5	0/5	
	Feed	0.5	5									0/5	0/5	10^0.8^SID_50_/g
		5	5									0/5	0/5	
		50	5				1					1/5	1/5	
		100	5				2					2/5	2/5	
		250	5		2	1						3/5	3/5	
		500	5		1		2	1				4/5	4/5	
3	Intramuscular	10^−6^	5		2	3						5/5	5/5	10^8.7^SID_50_/g
		10^−7^	5			2	2					4/5	4/5	
		10^−8^	5									0/5	0/5	
		10^−9^	5									0/5	0/5	
	Peroral	10^0^	5				1	1				2/5	2/5	10^1.1^SID_50_/g
		10^−1^	5									0/5	0/5	
		10^−2^	5									0/5	0/5	
		10^−3^	5									0/5	0/5	
	Feed	0.5	5									0/5	0/5	10^0.8^SID_50_/g
		5	5									0/5	0/5	
		50	5					1				1/5	1/5	
		100	5					2				2/5	2/5	
		250	5				1	1				2/5	2/5	
		500	5				2	1	1			4/5	4/5	

Intramuscular injection, peroral inoculation and feeding (feed).

### Role of PM as intestinal barrier

#### Removal of PM by a peroral flush

**Figure 3:**
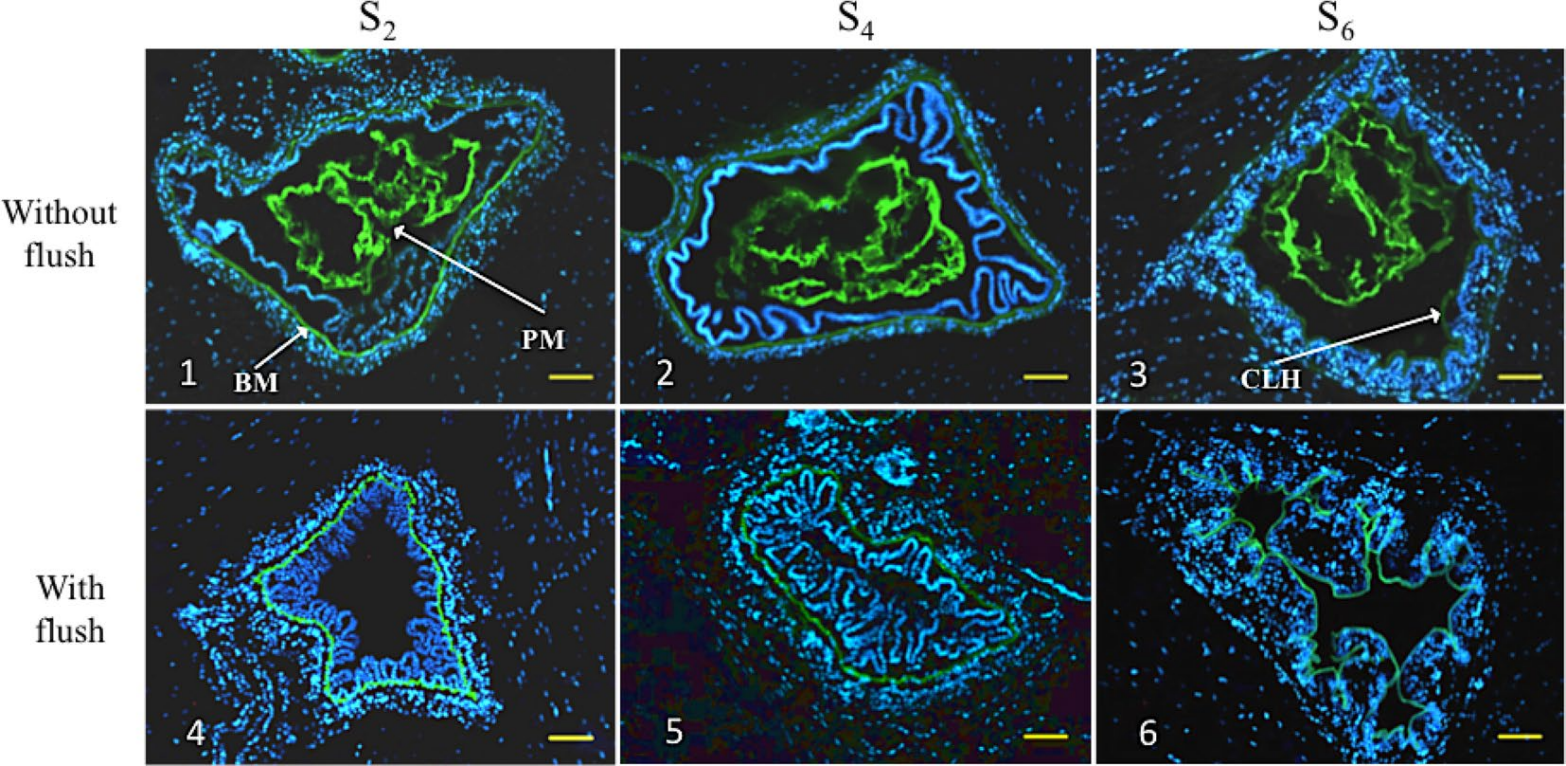
**Presence/absence of peritrophic membrane in the gut of shrimp without and with a peroral flush.** Peritrophic membrane (PM) was stained with FITC-linked succinylated (WGA) wheat germ agglutinin and analyzed by fluorescence microscopy. Bar = 100 µm. The cell nuclei were stained by Hoechst. Photomicrographs 1–3 show the presence of PM in the midgut (segment S_2_ and S_4_) and hindgut lumen (segment S_6_) of control samples (without a peroral flush). Photomicrographs 4–6 show the absence of PM in the midgut and hindgut lumen of perorally flushed shrimp. FITC-linked succinylated WGA also labeled the basement membrane (BM) of the midgut (photomicrographs 1, 2, 4 and 5) and the cuticle that lined the hindgut lumen (CLH, photomicrographs 3 and 6).

**Figure 4:**
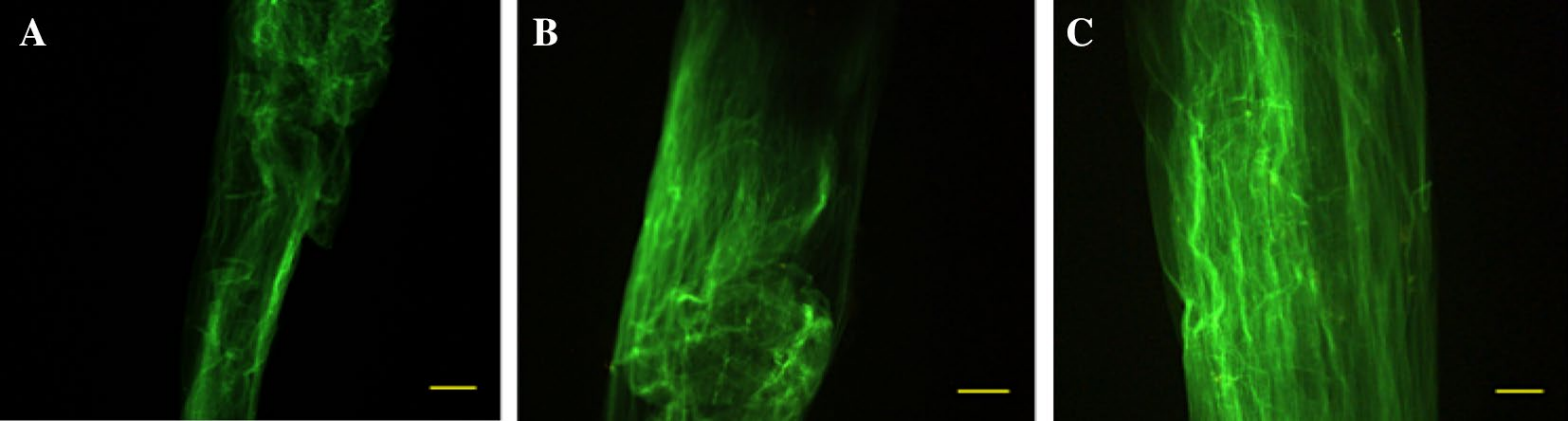
**FITC-WGA labeled peritrophic membrane enclosing a feed bolus, collected upon peroral flushing. A** Anterior region. **B** Middle region. **C** Posterior region. Bar = 100 µm.

Microscopical observation of cryosections showed that three structures were stained with fluorescein-linked succinylated WGA: the PM, the basement membrane (BM) and the cuticle that lined hindgut lumen (CLH, only in the hindgut). The PM was absent in the midgut (sections S_2_ and S_4_) and hindgut (section S_6_) in the shrimp that were flushed perorally, while the PM was clearly visible in the control samples at the 3 sampled segments (Figure 3; Additional file 1; Table 2). The PM in the midgut of intact control shrimp stained with FITC-WGA consisted of multiple layers (Figure 3; Additional file 1). The intensity of the FITC-WGA staining of the fecal material collected after flushing increased from the anterior to posterior position (Figure 4).

**Table 2:** **The presence of peritrophic membrane in** ***P. vannamei*** **shrimp without and with a peroral flush.**

Peroral flush	Molt stage	No. of shrimp	PM confirmed by FITC- WGA			No. of shrimp with the presence of PM
			S_2_	S_4_	S_6_	
No (Control)	B	3	+++	+++	+++	3/3
	C	3	+++	+++	+++	3/3
	D1	3	+++	+++	+++	3/3
	D2	3	+++	+++	+++	3/3
Yes	B	3	−−−	−−−	−−−	0/3
	C	3	−−−	−−−	−−−	0/3
	D1	3	−−−	−−−	−−−	0/3
	D2	3	−−−	−−−	−−−	0/3

Midgut sample sites (S_2_, S_4_), hindgut sample site (S_6_).

#### Effect of PM removal on WSSV infection upon peroral inoculation with WSSV

In the first experiment, among the groups of shrimp injected with dilutions 10^−6^, 10^−7^ and 10^−8^ of WSSV stock 2, all shrimp in dilution 10^−6^ and 3 out of 5 shrimp in dilution 10^−7^ died between 36 and 60 hpi. All other animals survived until the end of the experiment. When a peroral inoculation was performed in PM-intact shrimp with dilutions 10^0^, 10^−1^, 10^−2^ and 10^−3^ of the same WSSV stock, only 2 out of 5 shrimp of dilution 10^0^ died. In PM-negative shrimp, mortality was observed in 2 shrimp of dilution 10^0^ and 1 shrimp of dilution of 10^−1^. All other shrimp survived until the end of the experiment.

In the second experiment, among the groups of shrimp injected with 10^−6^, 10^−7^ and 10^−8^ of WSSV stock 2, all shrimp in dilution 10^−6^ and 4 out of 5 shrimp in dilution 10^−7^ died between 36 and 60 hpi. All other shrimp survived until the end of the experiment. When a peroral inoculation was performed in PM-intact shrimp with 10^0^, 10^−1^, 10^−2^ and 10^−3^ of WSSV stock 2, 1 out of 5 shrimp in dilution 10^0^, 1 in dilution 10^−1^, 1 in dilution 10^−2^ died between 36 and 60 hpi. Peroral inoculation of PM-negative shrimp induced mortality in 3 shrimp with dilution 10^0^ and 1 in dilution 10^−1^ between 48 and 60 hpi. Other shrimp survived until the end of the experiment.

In the third experiment, in the groups of shrimp injected with WSSV inoculum diluted 10^−6^–10^−8^, all shrimp in the dilution 10^−6^ and 12 out of 15 shrimp in the dilution 10^−7^ died between 36 and 84 hpi. Other shrimp survived until the end of the experiment. When shrimp were inoculated perorally with 10^0^, 10^−1^, 10^−2^ and 10^−3^ of WSSV stock 2, 5 deaths out of 15 PM-intact shrimp in dilution 10^0^ and 1 in dilution 10^−1^ were recorded between 36 and 60 hpi. Peroral inoculation in PM-negative shrimp caused mortality in 11 shrimp in dilution 10^0^ and 2 shrimp in dilution 10^−1^. All other shrimp survived until the end of the experiment at 120 hpi.

IIF analysis of cephalothorax of dead, moribund and surviving shrimp revealed that all dead and moribund animals were WSSV positive, while all surviving shrimp were WSSV negative. Analysis of midgut of dead and moribund shrimp showed that CT of the midgut of dead and moribund shrimp were WSSV positive, whereas the EC of the midgut were WSSV negative (Figure 2). The mean virus titers that were determined in the three experiments upon intramuscular injection and peroral inoculation of shrimp with and without PM were 10^8.6 ± 0.12^, 10^1.13 ± 0.06^ and 10^1.53 ± 0.21^ SID_50_/mL, respectively (Table 3). Compared with the intramuscular route, 10^7.5^ times more virus was needed to infect a PM-intact shrimp, while 10^7.1^ times more virus was necessary to infect a PM-negative shrimp via oral inoculation. The Chi square test on infection rates of shrimp showed that there was not a significant effect of removal of PM on the susceptibility of shrimp to WSSV infection (*p* > 0.05).

**Table 3:** **Infection titers of a WSSV stock in** ***P. vannamei*** **without and with removal of the peritrophic membrane.**

Experiment	Inoculation route	Removal of PM	Dilution tissue homogenate	No. of shrimp	Mortality at different time points (hpi)									Infection by IIF	Virus titer
					24	36	48	60	72	84	96	120	Total		
1	Intramuscular	No	10^−6^	5		2	2	1					5/5	5/5	10^8.5^ SID_50_/mL
		No	10^−7^	5			1	2					3/5	3/5	
		No	10^−8^	5									0/5	0/5	
	Peroral	No	10^0^	5			2						2/5	2/5	10^1.1^ SID_50_/mL
		No	10^−1^	5									0/5	0/5	
		No	10^−2^	5									0/5	0/5	
		No	10^−3^	5									0/5	0/5	
		Yes	10^0^	5		1	1						2/5	2/5	10^1.3^ SID_50_/mL
		Yes	10^−1^	5						1			1/5	1/5	
		Yes	10^−2^	5									0/5	0/5	
		Yes	10^−3^	5									0/5	0/5	
2	Intramuscular	No	10^−6^	5		2	1	2					5/5	5/5	10^8.7^ SID_50_/mL
		No	10^−7^	5			2	2					4/5	4/5	
		No	10^−8^	5									0/5	0/5	
	Peroral	No	10^0^	5		1							1/5	1/5	10^1.2^ SID_50_/mL
		No	10^−1^	5				1					1/5	1/5	
		No	10^−2^	5				1					1/5	1/5	
		No	10^−3^	5									0/5	0/5	
		Yes	10^0^	5			1	2					3/5	3/5	10^1.6^ SID_50_/mL
		Yes	10^−1^	5			1						1/5	1/5	
		Yes	10^−2^	5									0/5	0/5	
		Yes	10^−3^	5									0/5	0/5	
3	Intramuscular	No	10^−6^	15		2	4	4	4	1			15/15	15/15	10^8.7^ SID_50_/mL
		No	10^−7^	15		1	2	4	4	1			12/15	12/15	
		No	10^−8^	15									0/15	1/15	
	Peroral	No	10^0^	15		1	1	3					5/15	5/15	10^1.1^ SID_50_/mL
		No	10^−1^	15		1							1/15	1/15	
		No	10^−2^	15									0/15	0/15	
		No	10^−3^	15									0/15	0/15	
		Yes	10^0^	15		2	3	2	3	1			11/15	11/15	10^1.7^ SID_50_/mL
		Yes	10^−1^	15			2						2/15	2/15	
		Yes	10^−2^	15									0/15	0/15	
		Yes	10^−3^	15									0/15	0/15	

## Discussion

In vivo titration is generally used to define the infectivity of a WSSV stock [7, 18, 41]. In the present study, the infectivity of WSSV was first determined in *P. vannamei* by intramuscular injection and peroral inoculation. The results indicated that a homogenate of WSSV infected shrimp is highly infectious when directly injected into shrimp. In contrast, there are strong restrictions on the infection of shrimp via the digestive system even when the PM is removed. These findings were in conflict with earlier results from our group published by Escobedo-bonilla et al. [18]. In that paper, it was shown that shrimp could be easily infected upon peroral inoculation using a rigid plastic pipette tip (no. 790 004 Biozym). However, by using dye, we could demonstrate that the fluid was crossing the gastrointestinal tract and was entering the hemocoel. Therefore, we have changed the inoculation device. In the present study, we have used a softer and flexible 24G Surflo-W catheter. By the use of dye, it was demonstrated that peroral inoculation of shrimp with this 24G Surflo-W catheter is not damaging the gastrointestinal tract. The findings in the present study were also in contrast with other researchers who detected a 100% mortality by feeding infected shrimp [19, 43]. Different explanations can be forwarded. In the latter studies, animals were pooled in the same tank, fed with WSSV infected tissue shrimp for several days and terminated often after more than 5 days. It is very well possible that a larger amount of virus has been given with the feed and that cannibalism occurred when a few primarily infected shrimp became infected and died, resulting in large amounts of virus becoming homogeneously distributed in the water of the tanks. This may have triggered infection via waterborne route. However, our results are in agreement with the findings of others. Laramore reported that individual peroral feeding of *P. vannamei* with WSSV infected tissue at 10% of their body weight did not result in a 100% mortality [20]. Another study on peroral infection of *P. vannamei* with 200 µL of a WSSV inoculum, containing 10^7^ WSSV genome copies/mL, conducted by Gitterle et al. [17] also showed that cumulative mortality of shrimp was less than 100%.

In order to understand the factors determining virus infectivity via oral route, it is very important to have a good knowledge on the anatomy of the host digestive system. In decapod crustaceans, the digestive system is divided into three major regions: foregut, midgut, and hindgut. The foregut is composed of the mouth, esophagus, cardiac and pyloric stomach chambers, which are covered with a cuticle layer. The food ingested via the mouth moves through the esophagus and ends into the cardiac stomach chamber. Through the process of cutting, crushing, mixing by the lateral teeth systems and filtering by a cardiac setal screen in the cardiac chamber, the processed food is drained into the pyloric chamber. In the pyloric region, the processed material is further sorted by the ampullary setal screen of gland filters into a liquid form for further digestion in the hepatopancreas and particles for subsequent transport into the midgut region [44–46]. The midgut region secretes the peritrophic membrane which wraps the material coming from the pyloric chamber [47, 48]. The hindgut is a simple cuticle lined tube, that functions in expelling the peritrophic membrane, containing the feces [49, 50]. In several species of crustaceans, the mesh size of the ampullary setal screen is estimated to be smaller than 100 nm [51, 52]. The pore size of midgut peritrophic membrane can be as small as 20 nm [47]. From these data and the size of WSSV of 70–380 nm [53], it is likely that if the internal barriers of cuticle and peritrophic membrane are not ruptured, WSSV can not reach the epithelial cells. That is why, in the present study, it was examined if removal of the PM could facilitate the infection of the underlying epithelial cells. In the present study, N-acetyl-D-glucosamine of the PM was stained with FITC-WGA and staining of cryosections of midgut and feces revealed that the PM of *P. vannamei* is multilayered, which is similar to what has been described in penaeid shrimp by Wang et al. [48] and Martin et al. [47]. The authors described that *P. vannamei* possesses a type I of PM, that is continuously formed on the surface of epithelial midgut cells and consists of three stages: PM in stage 1 and 2 is closely attached to the microvilli of epithelial midgut cells, and PM in stage 3 is detached. After the removal of the PM by a peroral flush and directly thereafter the peroral inoculation of WSSV suspension, it was observed that the inoculum was filling the complete gastrointestinal tract (Additional file 2). Therefore, the virus had direct access to the epithelial cells. Within this short time frame, the epithelial cells were not able to produce a new PM. However, this PM removal did not facilitate WSSV infection of the underlying epithelial cells. This observation was in agreement with that found by Arts [54]. The author reported that nuclei of epithelial midgut cells of WSSV-infected *P. monodom* were negative with WSSV by VP28-immunoreactive and electron microscopy study. This is indicative for a state of resistance of epithelial cells to infection and the absence of receptors on the luminal surface of these cells. In addition, the infectivity of WSSV in shrimp via peroral infection may be largely decreased by digestive enzymes. This impact could be similar to the one described for insects [55]. Another factor that may limit the penetration of WSSV through shrimp midgut is the basement membrane, a firm layer of connective tissue underneath the epithelial cells. In insects, the basement membrane is well known to prevent virus entry into the hemocoel [31, 56, 57]. In the present study, the basement membrane could be visualized by FITC-WGA which is in agreement with the finding of Martin et al. [47]. When one considers all barriers and viral unfriendly environmental factors that WSSV encounters in the digestive system of shrimp, it is easy to understand why WSSV is poorly infectious for shrimp via peroral route.

In conclusion, the present study revealed that a homogenate of WSSV infected shrimp is highly infectious when injected into shrimp, whereas it is poorly infectious via oral route. Removal of the PM slightly but not significantly facilitated the infection of shrimp. From these findings, it is highly questioned if per os uptake of WSSV is the major route of spread for the virus. More work needs to be done on finding more important portals of entry for WSSV in shrimp which will finally lead to the development of more effective WSSV control measures.

## Additional files

10.1186/s13567-016-0321-5 **Detection of peritrophic membrane in the gut of shrimp without and with a peroral flush**. Peritrophic membrane was stained with FITC-linked succinylated WGA wheat germ agglutinin, cell nuclei with Hoechst and analyzed by fluorescence microscopy. Bar = 100 μm. Photomicrographs 1 to 36 show the presence of PM in the midgut (segment S2 and S4) and hindgut (segment S6) of control samples (without a peroral flush). Photomicrographs 37 to 72 show the absence of PM in the midgut and hindgut of perorally flushed shrimp. Code explanation: S1-B-S2; S1: shrimp number 1, B: shrimp in stage B of the molt cycle, S2: cross-section at segment 2.
10.1186/s13567-016-0321-5 **Presence of virus inoculum in the midgut of shrimp after a peroral inoculation**. (1) Before a per os flush to remove the PM, the midgut (MG) of shrimp was visible in the back of the animal. (2) After a per os flush, the PM was coming out of the anus. (3) Afterwards, the shrimp was inoculated with 50 μL of WSSV suspension (VS), directly the removal of the PM. The inoculum was present all over the gastrointestinal tract as can be seen in the figure.

## Abbreviations

WSSV: white spot syndrome virus
PM: peritrophic membrane
SID_50_: shrimp infectious dose 50% endpoint
ARC: Artemia Reference Center
MOETVIED: Vietnamese Ministry of Education and Training
DABCO: diaza-bicyclo-octane
MBW: mean body weight
IIF: indirect immunofluorescence
SPF: specific pathogen free
FITC-WGA: fluorescein isothiocyanate-Wheat germ agglutinin
Rpm: rounds per minute
PBS: phosphate-buffered saline
TSV: taura syndrome virus
YHV: yellow head virus
IHHNV: infectious hypodermal and hematopoietic necrosis virus
PL: post-larvae
BM: basement membrane
CLH: cuticle lined hindgut
FEC: folded epithelial cells

## References

[1] Chou H-Y, Huang C-Y, Wang C-H, Chiang H-C, Lo C-F (1995). Pathogenicity of a baculovirus infection causing white spot syndrome in cultured penaeid shrimp in Taiwan. Dis Aquat Organ.

[2] Escobedo-Bonilla C, Alday-Sanz V, Wille M, Sorgeloos P, Pensaert M, Nauwynck H (2008). A review on the morphology, molecular characterization, morphogenesis and pathogenesis of white spot syndrome virus. J Fish Dis.

[3] Durand S, Lightner D, Nunan L, Redman R, Mari J, Bonami J (1996). Application of gene probes as diagnostic tools for white spot baculovirus (WSBV) of penaeid shrimp. Dis Aquat Organ.

[4] Pradeep B, Shekar M, Karunasagar I, Karunasagar I (2008). Characterization of variable genomic regions of indian white spot syndrome virus. Virology.

[5] Sánchez-Paz A (2010). White spot syndrome virus: an overview on an emergent concern. Vet Res.

[6] Soowannayan C, Phanthura M (2011). Horizontal transmission of white spot syndrome virus (WSSV) between red claw crayfish *cherax quadricarinatus* and the giant tiger shrimp *Penaeus monodon*. Aquaculture.

[7] Corteel M, Dantas-Lima JJ, Wille M, Alday-Sanz V, Pensaert MB, Sorgeloos P, Nauwynck HJ (2009). Molt stage and cuticle damage influence white spot syndrome virus immersion infection in penaeid shrimp. Vet Microbiol.

[8] Prior S, Browdy CL, Shepard EF, Laramore R, Parnell PG (2003). Controlled bioassay systems for determination of lethal infective doses of tissue homogenates containing taura syndrome or white spot syndrome virus. Dis Aquat Organ.

[9] Soto MA, Lotz JM (2001). Epidemiological parameters of white spot syndrome virus infections in *L. vannamei* and *L*. Setiferus. J Invertebr Pathol.

[10] Tuyen N, Verreth J, Vlak J, de Jong M (2014). Horizontal transmission dynamics of white spot syndrome virus by cohabitation trials in juvenile *P. monodon* and *P. vannamei*. Prev Vet Med.

[11] Lo C-F, Ho C-H, Chen C-H, Liu K-F, Chiu Y-L, Yeh P-Y, Peng S-E, Hsu H-C, Liu H-C, Chang C-F, Su M-S, Wang C-H, Kou G-H (1997). Detection and tissue tropism of white spot syndrome baculovirus (WSBV) in captured brooders of *Penaeus monodon* with a special emphasis on reproductive organs. Dis Aquat Organ.

[12] Rahman M, Escobedo-Bonilla C, Corteel M, Dantas-Lima J, Wille M, Sanz VA, Pensaert M, Sorgeloos P, Nauwynck H (2006). Effect of high water temperature (33 °C) on the clinical and virological outcome of experimental infections with white spot syndrome virus (WSSV) in specific pathogen-free (SPF) Litopenaeus vannamei. Aquaculture.

[13] Tendencia EA, Bosma RH, Usero RC, Verreth JA (2010). Effect of rainfall and atmospheric temperature on the prevalence of the whitespot syndrome virus in pond-cultured Penaeus monodon. Aquaculture.

[14] Peinado-Guevara LI, López-Meyer M (2006). Detailed monitoring of white spot syndrome virus (WSSV) in shrimp commercial ponds in sinaloa, mexico by nested PCR. Aquaculture.

[15] Gunalan B, Soundarapandian P, Dinakaran G (2010). The effect of temperature and ph on wssv infection in cultured marine shrimp *Penaeus monodon* (Fabricius). Middle East J Sci Res.

[16] Vidal OM, Granja CB, Aranguren F, Brock JA, Salazar M (2001). A profound effect of hyperthermia on survival of *Litopenaeus vannamei* juveniles infected with white spot syndrome virus. J World Aquac Soc.

[17] Gitterle T, Gjerde B, Cock J, Salazar M, Rye M, Vidal O, Lozano C, Erazo C, Salte R (2006). Optimization of experimental infection protocols for the estimation of genetic parameters of resistance to white spot syndrome virus (WSSV) in *Penaeus litopenaeus vannamei*. Aquaculture.

[18] Escobedo-Bonilla C, Audoorn L, Wille M, Sanz VA, Sorgeloos P, Pensaert M (2006). Standardized white spot syndrome virus (WSSV) inoculation procedures for intramuscular or oral routes. Dis Aquat Organ.

[19] Lightner D, Hasson K, White B, Redman R (1998). Experimental infection of western hemisphere penaeid shrimp with asian white spot syndrome virus and Asian yellow head virus. J Aquat Anim Health.

[20] Laramore SE (2007). Susceptibility of the peppermint shrimp *Lysmata wurdemanni* to the white spot syndrome virus. J Shellfish Res.

[21] Hasson K, Fan Y, Reisinger T, Venuti J, Varner P (2006). White-spot syndrome virus (WSSV) introduction into the gulf of mexico and texas freshwater systems through imported, frozen bait-shrimp. Dis Aquat Organ.

[22] Pérez F, Volckaert FA, Calderón J (2005). Pathogenicity of white spot syndrome virus on postlarvae and juveniles of penaeus *Litopenaeus vannamei*. Aquaculture.

[23] Hegedus D, Erlandson M, Gillott C, Toprak U (2009). New insights into peritrophic matrix synthesis, architecture, and function. Annu Rev Entomol.

[24] Wang P, Granados RR (2000). Calcofluor disrupts the midgut defense system in insects. Insect Biochem Mol Biol.

[25] Rao R, Fiandra L, Giordana B, de Eguileor M, Congiu T, Burlini N, Arciello S, Corrado G, Pennacchio F (2004). AcMNPV ChiA protein disrupts the peritrophic membrane and alters midgut physiology of Bombyx mori larvae. Insect Biochem Mol Biol.

[26] Plymale R, Grove MJ, Cox-Foster D, Ostiguy N, Hoover K (2008). Plant-mediated alteration of the peritrophic matrix and baculovirus infection in lepidopteran larvae. J Insect Physiol.

[27] Mitsuhashi W, Kawakita H, Murakami R, Takemoto Y, Saiki T, Miyamoto K, Wada S (2007). Spindles of an entomopoxvirus facilitate its infection of the host insect by disrupting the peritrophic membrane. J Virol.

[28] Takemoto Y, Mitsuhashi W, Murakami R, Konishi H, Miyamoto K (2008). The n-terminal region of an entomopoxvirus fusolin is essential for the enhancement of peroral infection, whereas the c-terminal region is eliminated in digestive juice. J Virol.

[29] Huber M, Cabib E, Miller LH (1991). Malaria parasite chitinase and penetration of the mosquito peritrophic membrane. Proc Natl Acad Sci U S A.

[30] Langer RC, Vinetz JM (2001). plasmodium ookinete-secreted chitinase and parasite penetration of the mosquito peritrophic matrix. Trends Parasitol.

[31] Martin GG, Rubin N, Swanson E (2004). Vibrio parahaemolyticus and *V. harveyi* cause detachment of the epithelium from the midgut trunk of the penaeid shrimp *Sicyonia ingentis*. Dis Aquat Organ.

[32] Tran L, Nunan L, Redman RM, Mohney LL, Pantoja CR, Fitzsimmons K, Lightner DV (2013). Determination of the infectious nature of the agent of acute hepatopancreatic necrosis syndrome affecting penaeid shrimp. Dis Aquat Organ.

[33] Priya T, Li F, Zhang J, Wang B, Zhao C, Xiang J (2009). Molecular characterization and effect of RNA interference of retinoid X receptor (RXR) on E75 and chitinase gene expression in Chinese shrimp *Fenneropenaeus chinensis*. Comp Biochem Physiol B Biochem Mol Biol.

[34] Tan SH, Degnan BM, Lehnert SA (2000). The *Penaeus monodon* chitinase 1 gene is differentially expressed in the hepatopancreas during the molt cycle. Mar Biotechnol.

[35] Proespraiwong P, Tassanakajon A, Rimphanitchayakit V (2010). Chitinases from the black tiger shrimp *Penaeus monodon*: phylogenetics, expression and activities. Comp Biochem Physiol B Biochem Mol Biol.

[36] Peters G, Saborowski R, Buchholz F, Mentlein R (1999). Two distinct forms of the chitin-degrading enzyme N-acetyl-β-d-glucosaminidase in the Antarctic krill: specialists in digestion and moult. Mar Biol.

[37] Robertson L, Bray W, Leung-Trujillo J, Lawrence A (1987). Practical molt staging of *Penaeus setiferus* and *Penaeus stylirostris*. J World Aquac Soc.

[38] Chan S-M, Rankin SM, Keeley LL (1988). Characterization of the molt stages in *Penaeus vannamei*: setogenesis and hemolymph levels of total protein, ecdysteroids, and glucose. Biol Bull.

[39] Corteel M, Dantas-Lima J, Wille M, Alday-Sanz V, Pensaert M, Sorgeloos P, Nauwynck H (2012). Moult cycle of laboratory-raised *Penaeus (litopenaeus) vannamei* and *P. monodon*. Aquacult Int.

[40] Jiravanichpaisal P, Bangyeekhun E, Söderhall K, Söderhall I (2001). Experimental infection of white spot syndrome virus in freshwater crayfish *Pacifastacus leniusculus*. Dis Aquat Organ.

[41] Escobedo-Bonilla C, Wille M, Sanz VA, Sorgeloos P, Pensaert M, Nauwynck H (2005). In vivo titration of white spot syndrome virus (WSSV) in specific pathogen-free *Litopenaeus vannamei* by intramuscular and oral routes. Dis Aquat Organ.

[42] Reed LJ, Muench H (1938). A simple method of estimating fifty per cent endpoints. Am J Epidemiol.

[43] Wang Q, White BL, Redman RM, Lightner DV (1999). Per os challenge of *Litopenaeus vannamei* postlarvae and *Farfantepenaeus duorarum* juveniles with six geographic isolates of white spot syndrome virus. Aquaculture.

[44] Felgenhauer BE, Abele LG (1985). Feeding structures of two atyid shrimps, with comments on caridean phylogeny. J Crustacean Biol.

[45] Lin F-Y (1996). Structure of the gland tillers in the pyloric stomach of *Penaeus japonicus* (Decapoda: Penaeidae). J Crustacean Biol.

[46] McGaw IJ, Curtis DL (2013). A review of gastric processing in decapod crustaceans. J Comp Physiol B.

[47] Martin GG, Simcox R, Nguyen A, Chilingaryan A (2006). Peritrophic membrane of the penaeid shrimp *Sicyonia ingentis*: structure, formation, and permeability. Biol Bull.

[48] Wang L, Li F, Wang B, Xiang J (2012). Structure and partial protein profiles of the peritrophic membrane (PM) from the gut of the shrimp *Litopenaeus vannamei*. Fish Shellfish Immunol.

[49] Barker P, Gibson R (1978). Observations on the structure of the mouthparts, histology of the alimentary tract, and digestive physiology of the mud crab *Scylla serrata* (Forskål) (Decapoda: Portunidae). J Exp Mar Biol Ecol.

[50] Dall W, Moriarty D, Mantel LH (1983). Functional aspects of nutrition and digestion. The biology of Crustacea.

[51] Kunze J, Anderson D (1979). Functional morphology of the mouthparts and gastric mill in the hermit crabs *Clibanarius taeniatus* (milne edwards), *Clibanarius virescens* (krauss), *Paguristes squamosus* McCulloch and *Dardanus setifer* (milne-edwards) (anomura: Paguridae). Aust J Mar Fresh Res.

[52] Ngoc-Ho N (1984). The functional anatomy of the foregut of *Porcetlana platychetes* and a comparison with *Galathea squamifera* and *Upogebia deltaura* (Crustacea: Decapoda). J Zool.

[53] Wang C-H, Lo C-F, Leu J-H, Chou C-M, Yeh P-Y, Chou H-Y, Tung M-C, Chang C-F, Su M-S, Kou G-H (1995). Purification and genomic analysis of baculovirus associated with white spot syndrome (WSBV) of *Penaeus monodon*. Dis Aquat Organ.

[54] Arts JA (2007). Haemocyte reactions in wssv immersion infected *Penaeus monodon*. Fish Shellfish Immunol.

[55] Brackney DE, Foy BD, Olson KE (2008). The effects of midgut serine proteases on dengue virus type 2 infectivity of *Aedes aegypti*. Am J Trop Med Hyg.

[56] Mellon D, Harrison FW, Humes AG (1992). Connective tissue and supporting structures. Microscopic anatomy of invertebrates.

[57] Passarelli AL (2011). Barriers to success: how baculoviruses establish efficient systemic infections. Virology.

