# Supplementary figures and images for: Per os infectivity of white spot syndrome virus (WSSV) in white-legged shrimp (Litopenaeus vannamei) and role of peritrophic membrane
Source: Vet Res. 2016 Feb 29;47:39. doi: 10.1186/s13567-016-0321-5 (PMC4772295; doi:10.1186/s13567-016-0321-5)

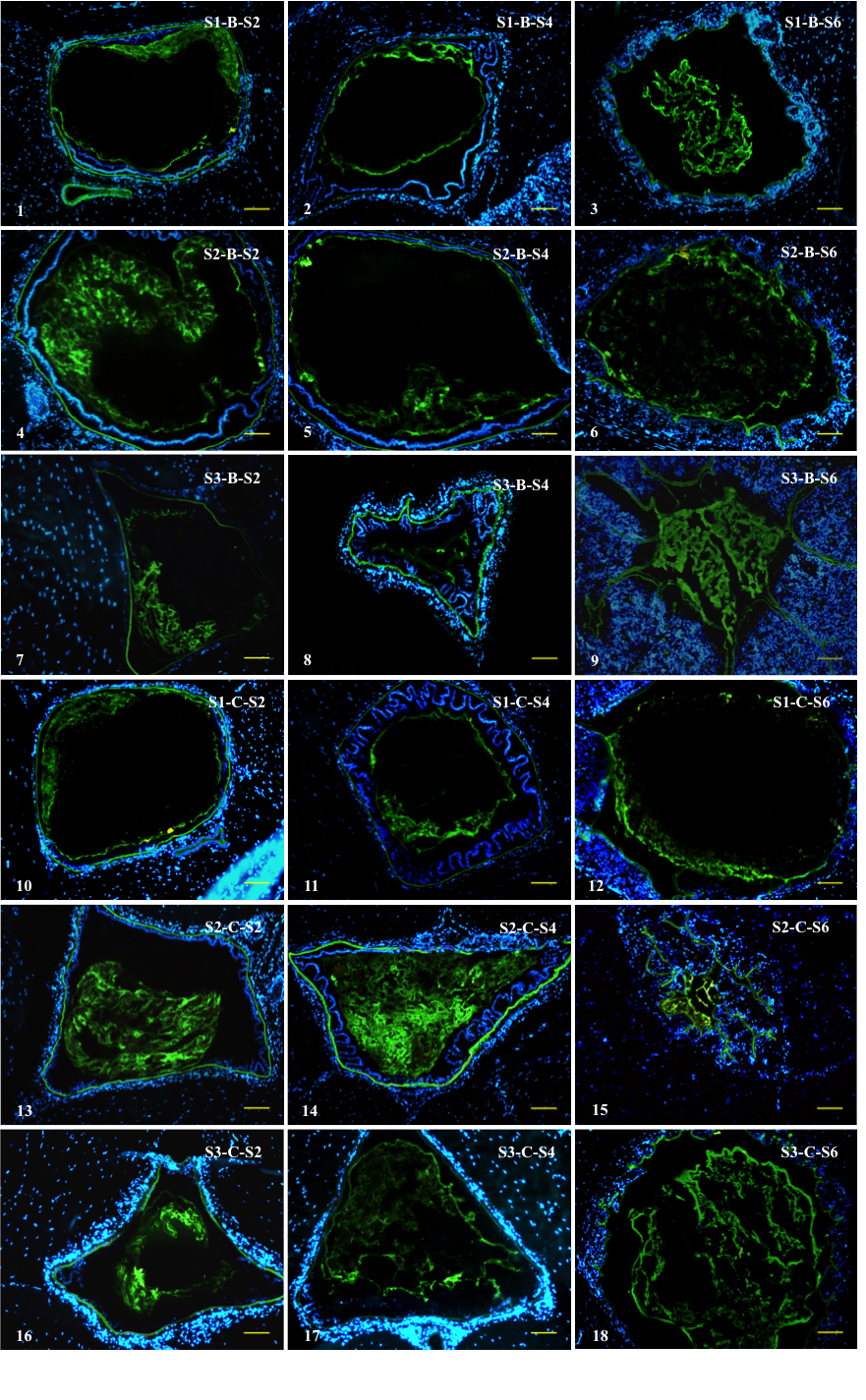


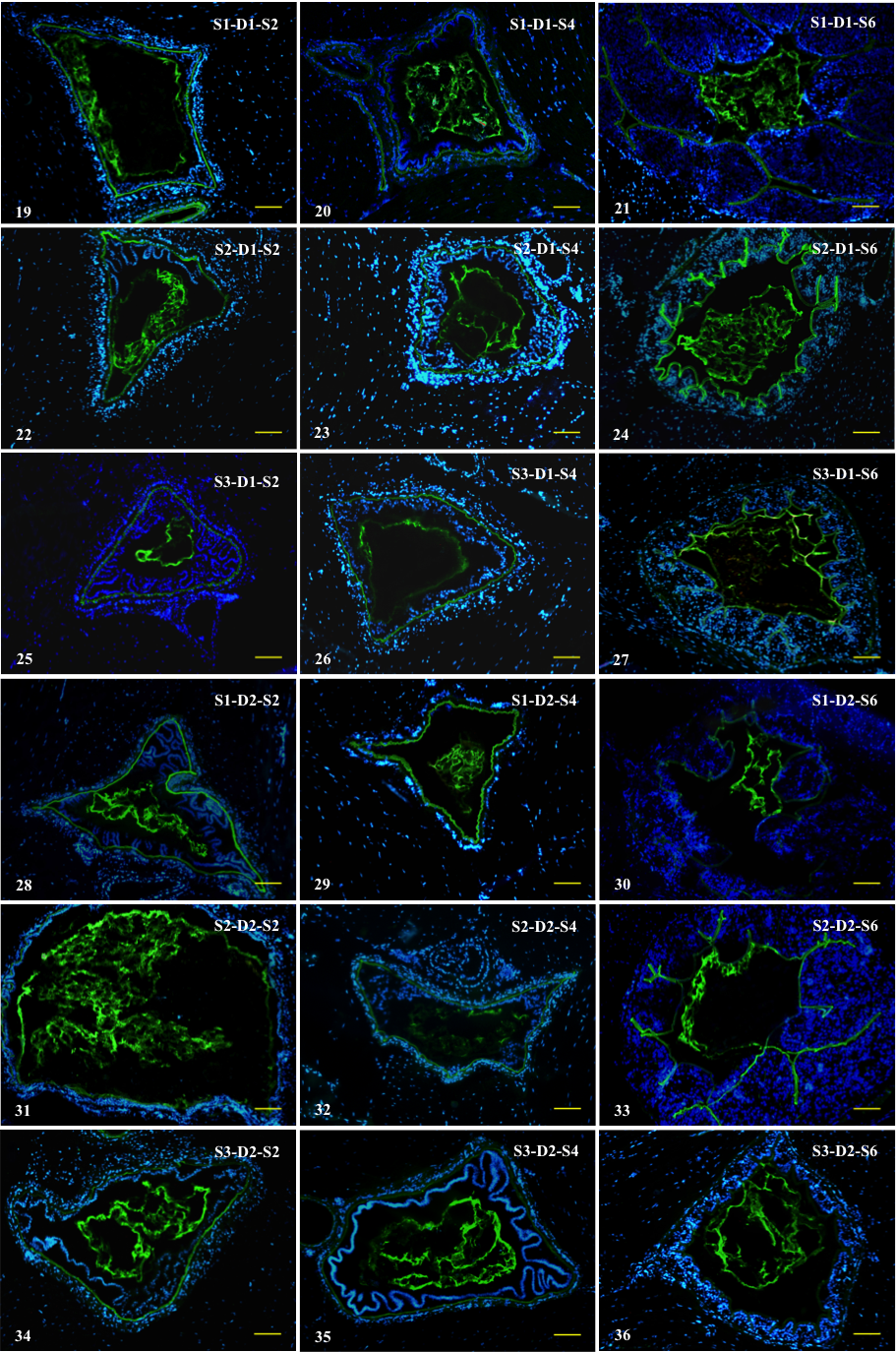


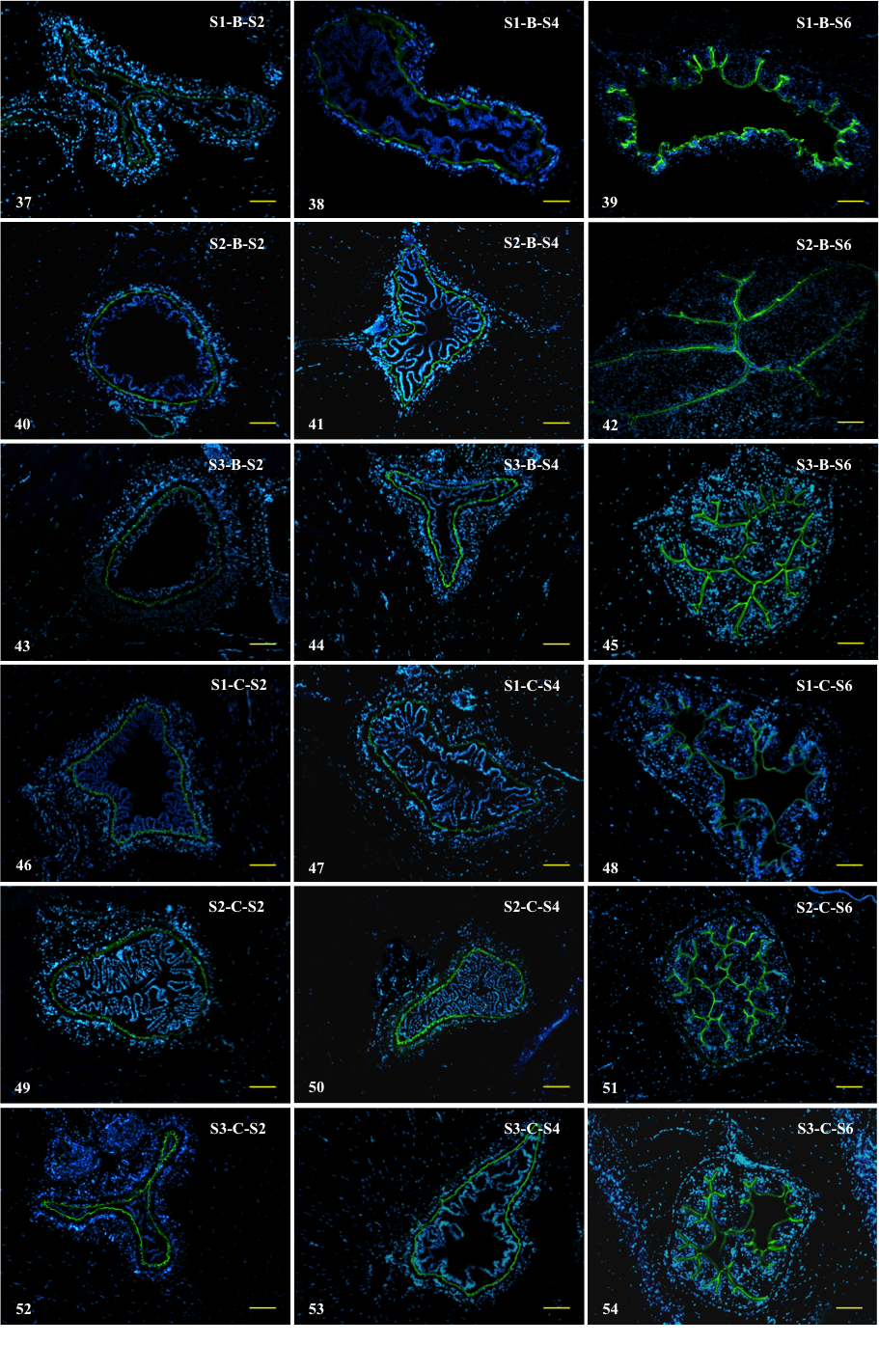


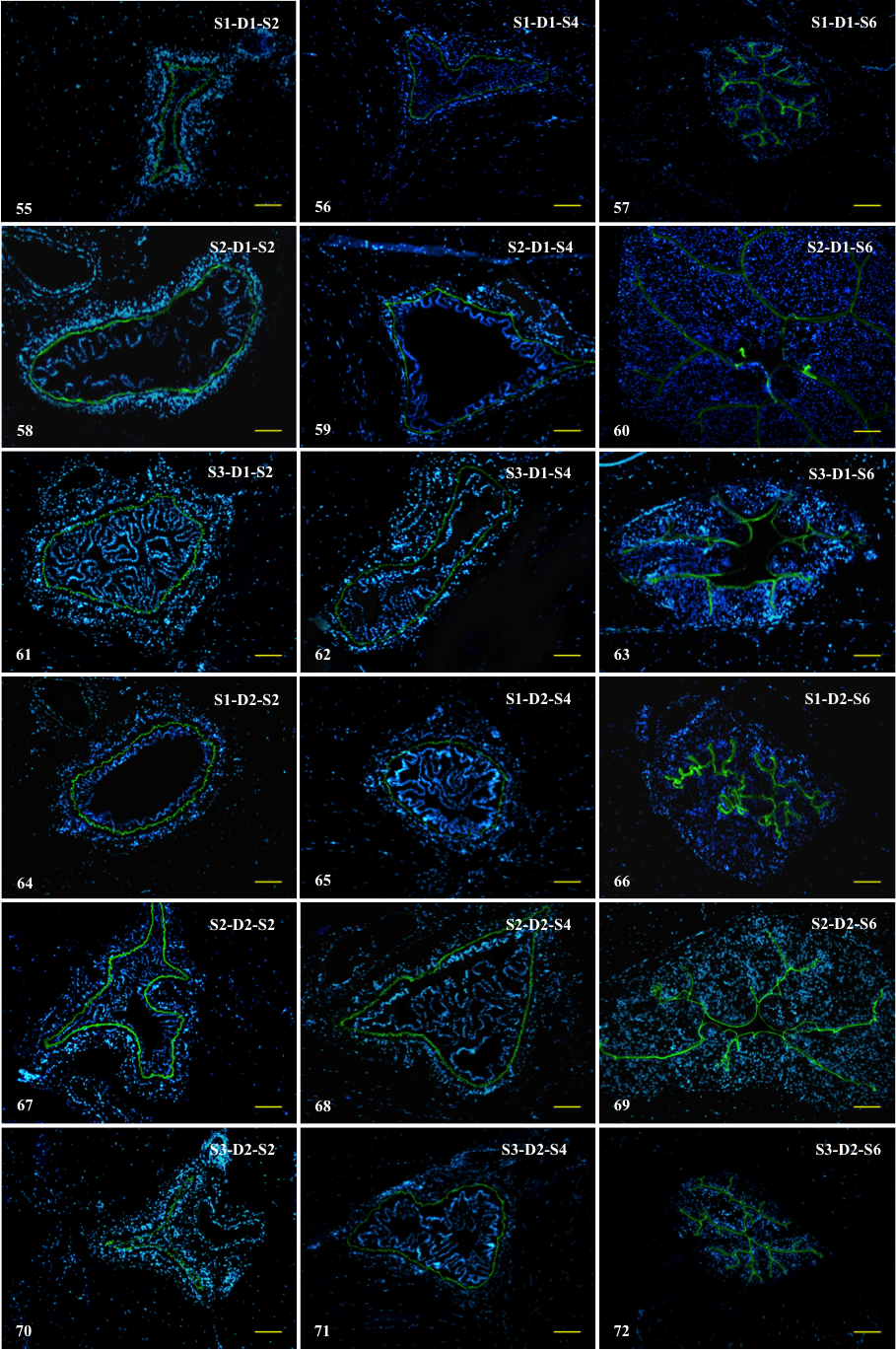

Supplement: Supplementary file 1 — 10.1186/s13567-016-0321-5 Detection of peritrophic membrane in the gut of shrimp without and with a peroral flush. Peritrophic membrane was stained with FITC-linked succinylated WGA wheat germ agglutinin, cell nuclei with Hoechst and analyzed by fluorescence microscopy. Bar = 100 μm. Photomicrographs 1 to 36 show the presence of PM in the midgut (segment S2 and S4) and hindgut (segment S6) of control samples (without a peroral flush). Photomicrographs 37 to 72 show the absence of PM in the midgut and hindgut of perorally flushed shrimp. Code explanation: S1-B-S2; S1: shrimp number 1, B: shrimp in stage B of the molt cycle, S2: cross-section at segment 2. [file 13567_2016_321_MOESM1_ESM.docx]

**
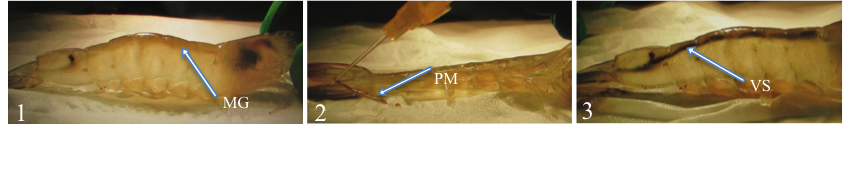
**

Supplement: Supplementary file 2 — 10.1186/s13567-016-0321-5 Presence of virus inoculum in the midgut of shrimp after a peroral inoculation. (1) Before a per os flush to remove the PM, the midgut (MG) of shrimp was visible in the back of the animal. (2) After a per os flush, the PM was coming out of the anus. (3) Afterwards, the shrimp was inoculated with 50 μL of WSSV suspension (VS), directly the removal of the PM. The inoculum was present all over the gastrointestinal tract as can be seen in the figure. [file 13567_2016_321_MOESM2_ESM.docx]
